# 

**Published:** 1924-02

**Authors:** 


New Series. Vol. III. No. 29 (Vol. LXXIII. No. 1871). February, 1924. Monthly, Price 6d. (POST FREE 7½d.)

